# Global Research on Neuropathic Pain Rehabilitation over the Last 20 Years

**DOI:** 10.1155/2021/5594512

**Published:** 2021-07-07

**Authors:** Xuan Su, Hao-Yu Hu, Chang Xu

**Affiliations:** ^1^Department of Sport Rehabilitation, Shanghai University of Sport, 399 Changhai Rd., Shanghai 200438, China; ^2^Department of Rehabilitation Medicine, Shanghai Shangti Orthopaedic Hospital, 188 Hengren Rd., Shanghai 200438, China; ^3^Department of Sport Psychology, Shanghai University of Sport, Shanghai 200438, China

## Abstract

**Background:**

Neuropathic pain has long been a very popular and productive field of clinical research. Neuropathic pain is difficult to cure radically because of its complicated etiology and uncertain pathogenesis. As pain worsens and persists, pain recovery techniques become more important, and medication alone is insufficient. No summary of bibliometric studies on neuropathic pain rehabilitation is yet available. The purpose of the present study is to analyze in a systematic manner the trends of neuropathic pain rehabilitation research over the period of 2000–2019.

**Methods:**

Studies related to neuropathic pain rehabilitation and published between January 2000 and December 2019 were obtained from the Science Citation Index-Expanded of Web of Science. No restrictions on language, literature type, or species were established. CiteSpace V and Microsoft Excel were used to capture basic information and highlights in the field.

**Results:**

Linear regression analysis showed that the number of publications on neuropathic pain rehabilitation significantly increased over time (*P* < 0.001). The United States showed absolute strength in terms of number of papers published, influence, and cooperation with other countries. Based on the subject categories of the Web of Science, “Rehabilitation” had the highest number of published papers (446), the highest number of citations (10,954), and the highest number of open-access papers (151); moreover, this category and “Clinical Neurology” had the same *H*-index (i.e., 52). “Randomized Controlled Trials” revealed the largest cluster in the cocitation map of references. The latest burst keywords included “Exercise” (2014–2019), “Functional Recovery” (2015–2019), and “Questionnaire” (2015–2019).

**Conclusion:**

This study provides valuable information for neuropathic pain rehabilitation researchers seeking fresh viewpoints related to collaborators, cooperative institutions, and popular topics in this field. Some new research trends are also highlighted.

## 1. Introduction

Neuropathic pain is a very popular and productive field of clinical research. In 2008, the IASP Special Interest Group (NeuPSIG) updated its definition of neuropathic pain as pain caused by a lesion or disease of the somatosensory system. Neuropathic pain is a fairly common disorder. Indeed, *Pain* reported that the best estimate of the prevalence of pain with neuropathic characteristics in the population may be between 6.9 and 10% [1–4]. Neuropathic pain is difficult to cure radically because of its complicated etiology and uncertain pathogenesis. This disease not only affects the quality of life and functions of patients but also increases the incidence of depression and anxiety, resulting in the wastage of medical resources and massive economic burdens [5, 6]. As pain worsens and persists, pain recovery techniques become more important, and medication alone is insufficient [7]. Psychosocial support and cognitive behavioral therapy may also be considered. Neuromodulation technology, minimally invasive technology, kinesiotherapy, traditional regimen, and multimodal management plans have shown good effects on pain management [8–14]. The rehabilitation of neuropathic pain is of great significance in addressing the symptoms and improving the clinical prognosis of patients [15].

No summary of the existing research on neuropathic pain rehabilitation is yet available. Bibliometrics combines mathematics, statistics, and philology to conduct quantitative research and analysis on a certain interdisciplinary field. It is an important academic link to obtain quantifiable, reproducible, and objective data [16]. In addition, bibliometrics can be used as a search tool to analyze the scope of impact of research findings and identify links between relevant and updated research, author networks, and institutions [17]. The Web of Science (WoS) is an online database of scientific citations that can be used to obtain data on citations, subjects, authors, institutions, and impact factors, thereby providing a useful search-and-analysis tool to generate representative data. The CiteSpace can be used to process and export search results directly to analyze published papers. Several articles on cancer rehabilitation, spinal cord injury (SCI) rehabilitation, traumatic brain injury rehabilitation, and total knee arthroplasty rehabilitation have been published [18–21]. This article mainly focused on the rehabilitation of neuropathic pain.

This review analyzes the current publications and development trends of neuropathic pain rehabilitation from the perspective of bibliometrics. The main institutions, extent of international cooperation, current situation, and trends are analyzed, and keyword cluster and world map analyses are used to reveal the research hot spots and leading countries in this field. A detailed bibliometric analysis of neuropathic pain rehabilitation research may help clinicians quickly and accurately classify and understand this field and guide future research directions.

## 2. Data and Methods

### 2.1. Data Collection

We collected synonyms related to neuropathic pain and rehabilitation and used “Subject Terms” for retrieval. The screening and downloading of literature for analysis was conducted on November 21, 2020. Literature from the last two decades (years 2000–2019) was downloaded from the Science Citation Index Expanded (SCI-Expanded) database of WoS.

Our search strategy was as follows: TS= (neuralgia∗ OR neurodynia∗ OR sciatica OR “nerve pain∗” OR “nerve cut” OR “nerve constriction” OR “nerve inflammation” OR “nerve crush” OR “nerve injury” OR “nerve ligation” OR “neuropathic pain” OR “peripheral neuropathy” OR “diabetic neuropathy” OR “chronic constriction injury”) AND TS= (“rehabilitation” or “physical medicine” or “physical therap∗” or “occupational therap∗”).

All of the data in this paper were extracted independently by the author (Xuan Su). EndNote X8 (Bld 7072, Thomson Research Soft, Stamford, CA, USA) and Microsoft Office Excel were used to extract the data to be downloaded from WoS. We strictly followed the established retrieval strategy, extracted the target literature collection, created the citation report, and then obtained the target data. Data on publication count, citation frequency (including self-citations), number of citations per year, number of citations in 2019, *H*-index, open-access papers, and essential science indicator (ESI) top papers were directly obtained data from WOS and used as bibliometric indicators for visual analysis. All of the relevant data and references were stored in text format for subsequent visualization analysis. Publication count refers to the quantitative contribution of an author or institution. The number of citations, which refers to the sum of citations of all items in a set, can indicate the average quality of published papers. The *H*-index, also known as the H-factor, was proposed by Hirsch. This index evaluates authors' academic achievements in a specific field [22, 23]. For instance, if the *H*-index of an author is 30, all papers published by the author have been cited at least 30 times in 30 papers. Higher *H*-index values indicate more influential and persuasive papers. Open-access papers refer to the number of publications whose peer-reviewed versions are available free of charge from a publisher's website or repository.

### 2.2. Inclusion Criteria

In this study, papers published in a wide variety of periodicals, including *Pain*, *Lancet*, and *Brain*, on neuropathic pain rehabilitation were included without restrictions on the type of article or language used. The types of literature mainly included articles, reviews, and proceedings. Both animal and clinical studies were included.

### 2.3. Statistical Analysis

The data were imported into CiteSpace (5.3.R11) in plain text format for analysis. CiteSpace V and Microsoft Excel were used to capture basic information and notable points in the field. The characteristics of the field were then studied in terms of discipline terms and keywords, and the publishing model of papers was assessed in terms of the number of publications in each country and the journal publishers. The frequency and percentage of journal and annual publications in each country were calculated on the basis of year of publication. The variation trends of research hot spots were studied through citation frequencies, keywords, and timeline views. Finally, we analyzed the citation trends of the top 10 countries, top 10 journals, and top 10 research fields to explore publishing patterns. IBM SPSS Statistics 22.0 (SPSS, Inc., Chicago, IL, USA) was used to calculate the number of changes and determine whether the data are statistically significant. Linear regression analysis was performed on the data using category as the dependent variable and year as the independent variable. For example, analyze the number of articles published each year. A *P* value of <0.05 was considered statistically significant.

## 3. Results

### 3.1. Publication Outputs and Growth Trend

A total of 1,518 papers conforming with the retrieval requirements were collected. Articles and reviews accounted for 94.1% of the total number of articles collected. The remaining literature types included non-article-type documents, including proceedings papers, editorial materials, meeting abstracts, book chapters, early-access articles, corrections, letters, and reprints.

**Figure 1:**
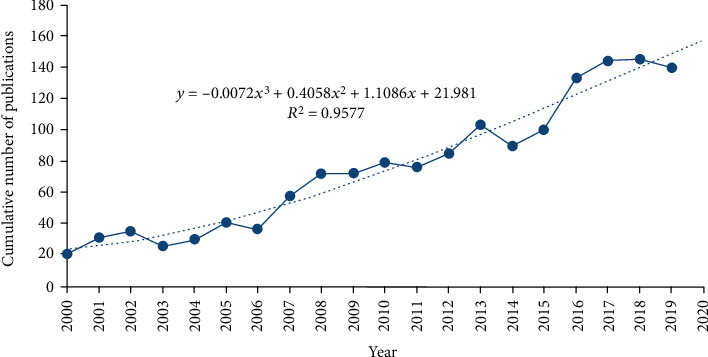
The number of annual publications on neuropathic pain rehabilitation research from 2000 to2019 and establish a time trend citation curve.

The annual publication volume generally increased with some fluctuations over the years (Figure 1). A statistically significant increase in number of papers published, from 21 articles in 2000 to 140 articles in 2019, was noted (*t* = 16.795, *P* < 0.001), thereby indicating that medical researchers are gradually expanding the field of research on the rehabilitation of neuropathic pain.

From 2000 to 2019, 1,518 papers were published in the field of neuropathic pain rehabilitation (average, 75 papers per year). The number of publications published in 2020 was forecasted on the basis of the growth rate curve of the number of publications by using the formular growth rate = −0.0072*x*^3^ + 0.4058*x*^2^ + 1.1086*x* + 21.981 (*R*^2^ = 0.9577), and the predicted number of papers to be published in 2020 was 158.

**Figure 2:**
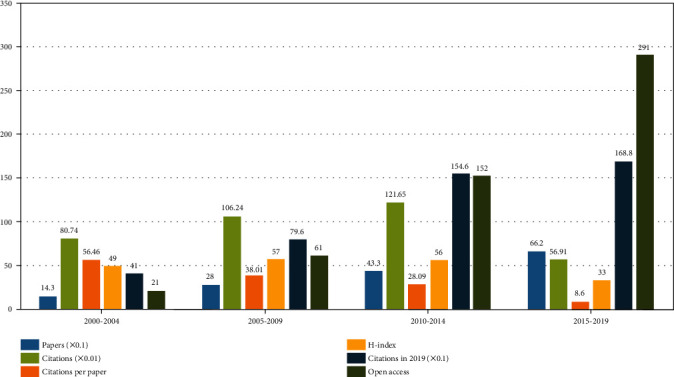
Number of papers, citations, citations per paper, open access paper, *H*-index, and citations in 2019 for each 5-year time period.

Among the four 5-year periods established (2000–2004, 2005–2009, 2010–2014, and 2015–2019), the most cited period per paper (5,646 times) was 2000–2004 (Figure 2), likely because this period represents the early stage of rehabilitation professional development, and only a small number of articles were available at the time. Although the number of articles published in this period was only 143, the total number of citations (8,074 times) in this period exceeded that in 2015–2019 (5,691 times). The *H*-index peaked from 2010 to 2014. The citations in 2019 and the number of open-access articles peaked from 2015 to 2019.

### 3.2. Distribution of National Geography and Institutions

**Figure 3:**
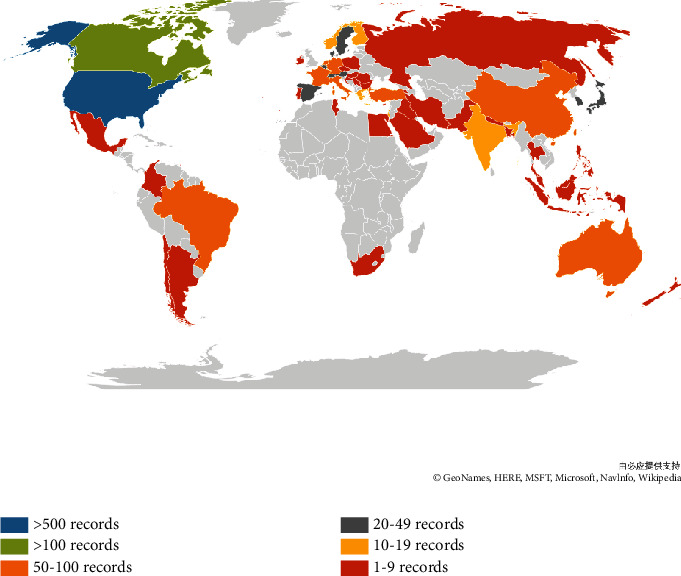
World map of total country output based on neuropathic pain rehabilitation research.

**Figure 4:**
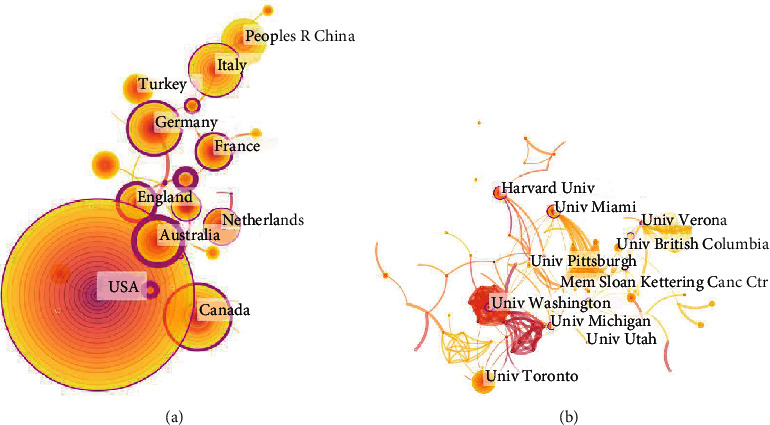
The analysis of countries and institutions. (a) Network map of countries/territories engaged in neuropathic pain rehabilitation research. (b) Network map of institutions engaged in neuropathic pain rehabilitation research.

Figure 3 shows a world map of all countries and territories in which studies on neuropathic pain rehabilitation had been published; here, the geographical distribution of publications covered 63 countries and territories.  Figure 4(a) shows the extensive cooperation between countries and regions.  Table 1 reveals the top 10 countries by number of published papers. The United States demonstrated a strong influence in this field, publishing the largest number of papers and five ESI top papers. Studies published in the United States also showed the largest number of citations (19,543 times), the highest *H*-index (71), and the greatest number of open-access articles (196).

Published papers in the field of neuropathic pain rehabilitation involved a total of 2,014 institutions.  Table 2 shows the top 10 institutions in terms of number of papers published. The papers of the Harvard University were cited the most (2,278 times), but the papers of the Mayo Clinic, the no. 1 hospital in the United States, were cited the most citations per year (112.85 times), with two ESI papers. The University of Toronto had the highest number of published papers (36), the University of Washington had the highest *H*-index (21), and the University of Pittsburgh had the highest number of open-access articles (15).  Figure 4(b) shows the degree of cooperation among the top 10 institutions engaged in neuropathic pain rehabilitation research. According to our analysis of countries and institutions, the Harvard University is the world's leading university in this field and the center of a cooperative network.

### 3.3. Analysis of the Top 10 Authors and Cocited Authors

A total of 1,518 papers on neuropathic pain rehabilitation research were written by 6,180 authors. Among the top 10 cocited authors (Table 3), Finnerup NB was cited 113 times, followed by Siddall PJ (95 times), and Dworkin RH (90 times). These authors are active and influential in the field of neuropathic pain rehabilitation. Vera Bril, from the Division of Neurology, Toronto General Hospital in Canada, studied the occurrence and development of diabetes and performed research on various possible neuropathies, including the clinical manifestations, diagnostic characteristics, and management of various sequelae [24–27]. Stefano Tamburin from the Department of Neuroscience, Biomedicine and Movement Sciences, University of Verona, found that the use of psychotherapy has a pain-relieving effect on neurological disorders. The author's team also demonstrated that different forms of psychological intervention measures, including cognitive behavior therapy, hypnosis, cognitive or behavioral techniques, mindfulness, acceptance and commitment therapy, brief interpersonal therapy, and virtual reality interventions, could effectively reduce the morbidity of different pains, such as musculoskeletal pain, fibromyalgia, central poststroke pain, phantom limb pain, pain secondary to SCI, diabetic neuropathy migraines and headaches, complex regional pain syndrome (CRPS), and medically unexplained symptoms [28–32]. In other words, pain is inextricably linked to cognition.

**Figure 5:**
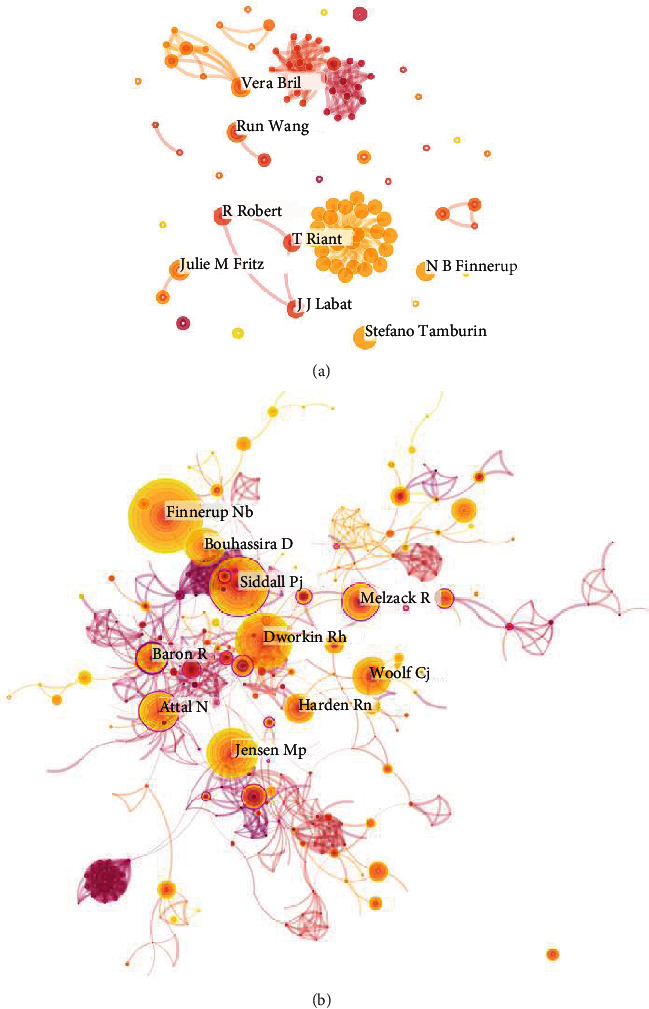
The analysis of authors. (a) Network map of active authors contributed to neuropathic pain rehabilitation research. (b) Network map of cocited authors contributed to neuropathic pain rehabilitation research.

Figure 5 illustrates author and cocited author cooperation maps. These two graphs provide effective and intuitive information that allows readers to observe the collaboration between authors. However, the centrality of cooperation at the author level is generally less than 0.03, thereby indicating that cooperation between researchers is not so close with certain limitations.

### 3.4. Analysis of the Top 10 Cocited References

References are an important component of high-quality papers that not only provides a strong argument for the author's findings but also expands the information chain and reflects the scientific value of research. In other words, references are an important index that reflects the scientific basis of a paper.

**Figure 6:**
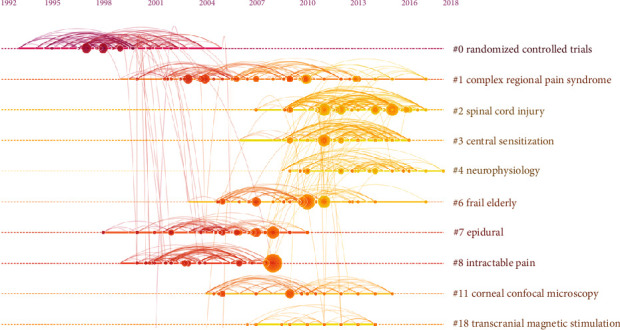
The analysis of references. Cocitation map (timeline view) of references from publications on neuropathic pain rehabilitation research.

A timeline view of the literature cocitation analysis is shown in Figure 6; here, active clusters named after the index terms cited in the literature are listed. Modularity, which is expressed as *Q* value, is a commonly used method to evaluate the strength of the network community structure. In this study, the *Q* value was 0.8705. A *Q* value higher than 0.3 indicates that the community structure is significant. The largest cluster (#0) was “randomized controlled trials,” followed by “complex regional pain syndrome” (#1), “spinal cord injury” (#2), and “central sensitization” (#3). Despite the fairly wide availability of research on the mechanisms involved in central sensitization or neuroinflammation in patients with chronic low back pain or musculoskeletal pain, the treatment of these issues remains a challenging scientific problem [33–37]. Experts recommend pain neuroscience education, cognitive behavioral therapy, and exercise therapy [38].

### 3.5. Bibliometric Analysis of the Journals

Over the last 20 years (2000–2019), a total of 585 journals published papers related to neuropathic pain rehabilitation. Active journals are defined as journals with the greatest number of publications within a certain period. As shown in Table 4, the top 10 active journals published 323 articles, accounting for 21.28% of the total number of published papers. Most journals have an impact factor ranging from 2 to 4. The journals with the highest and lowest impact factors were *Neurorehabilitation and Neural Repair* (IF 2019, 3.982) and *Journal of Rehabilitation Research and Development* (IF 2016, 1.277), respectively. Because of the diversity of categories, four journals remained in Q1, thereby indicating that the overall impact factor of published papers in this field is not high. However, some categories such as “rehabilitation” and “exercise science” could be found in Q1.

**Figure 7:**
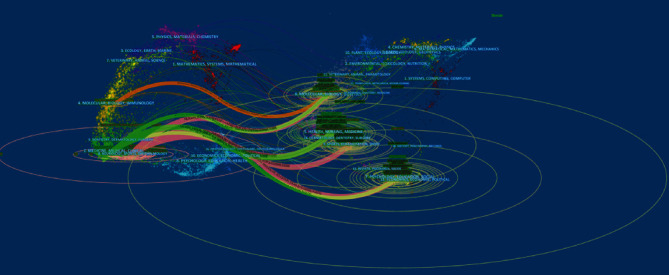
The dual-map overlay of journals related to on neuropathic pain rehabilitation research.

A dual-map overlay of journals is shown in Figure 7. The figure shows the disciplines covered by the journal in the form of labels. The colored line segment in the dual-map represents the cited connections, and the connection traces the citing journal to the cited journal. The number of authors is shown on the horizontal axis of the ellipse, whereas the number of publications is shown on the vertical axis of the ellipse. Most of the papers were published in journals dedicated to neurology, sports, and ophthalmology (left), and these journals were mostly cited by journals of psychology, education, and society (right).

### 3.6. Distribution of Keywords

**Figure 8:**
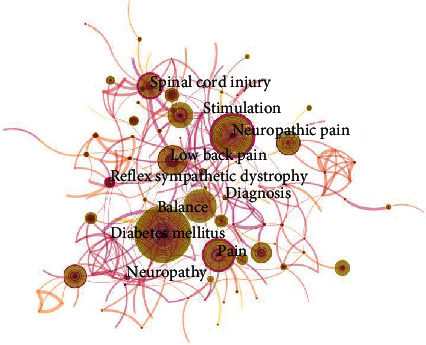
Network map of keyword cooccurrence in neuropathic pain rehabilitation research from 2000 to 2019.

Keywords are the core summary of a paper, which can be analyzed on the topic of the paper. Authors generally believe that the more frequently lexical pairs appear in the same literature, the closer the relationship between these two topics. The most common keywords were diabetes mellitus, neuropathic pain, low back pain, and spinal cord injury (as shown in Figure 8).

**Figure 9:**
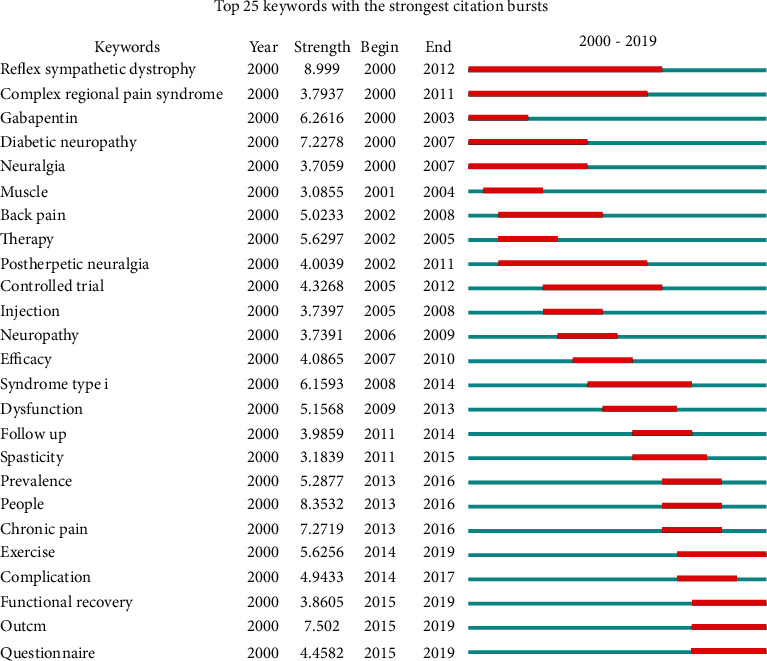
The keywords with the strongest citation bursts of publications on neuropathic pain rehabilitation research.

The frequent occurrence of keywords over a certain period of time is regarded as an indicator of frontier topics, vigorous development, and emerging trends. Keywords with the strongest citation bursts were derived from CiteSpace, and the results are shown in Figure 9. Here, the top 25 highlighted keywords from 2000 to 2019 are highlighted. Exercise, functional recovery, outcome, and questionnaire appeared to be newly developing research hot spots. The keyword “reflex sympathetic dystrophy,” which appeared only in 2012 had the highest citation burst rate (8.99).

### 3.7. Analysis of the Subject Categories

**Figure 10:**
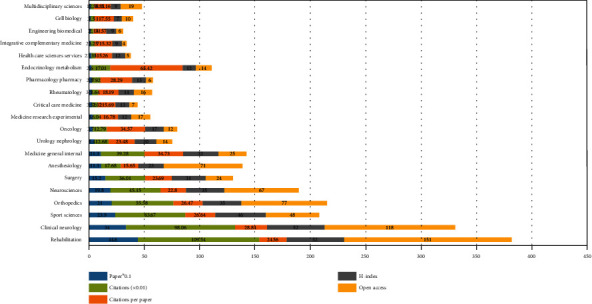
The number of papers, citations, citations per paper, open access papers, and *H*-index of the top 20 subject categories of the Web of Science.

The 1,518 papers on neuropathic pain rehabilitation research were allocated to 87 topic categories in WoS. Among the top 20 subject categories (Figure 10), “*Rehabilitation*” revealed the highest number of published papers (446), citations (10,954), and open-access papers (151). “*Anesthesiology*” showed the highest number of citations per item (26.64), and *Engineering Biomedical* demonstrated the highest number of citations per paper (65.42). The *H*-index of *Rehabilitation* and *Clinical Neurology* was identical at 52.

Linear regression analysis showed that the percentages statistically increased over time (*P* < 0.001) in the top 20 categories (*Rehabilitation*, *Clinical Neurology*, *Sport Sciences*, *Orthopedics*, *Neurosciences*, *Surgery*, *Anesthesiology*, *Medicine General Internal*, *Urology nephrology*, *Oncology*, *Medicine Research Experimental*, *Critical Care Medicine*, *Rheumatology*, *Pharmacology Pharmacy*, *Endocrinology Metabolism*, *Health Care Science Services*, *Integrative Complementary*, *Engineering Biomedical*, *Cell Biology*, and *Multidisciplinary Sciences*).

## 4. Discussion

### 4.1. Global Tendency of Neuropathic Pain Rehabilitation Research

In this review, the CiteSpace V software was used to carry out bibliometrics analysis in the field of neuropathic pain rehabilitation. Changes in bibliometric indicators, such as keywords, subject words, authors, countries, and institutions, over a time span of 20 years were then presented in diagrams and tables.

The output of publications showed a gradual annual increase (Figure 1). According to the number of papers published in the field of neuropathic pain rehabilitation in different countries as well as the overview of countries on the world map, the United States was relatively productive in this field of research (599), followed by Canada (113), China (92), and Germany (91). The top 10 countries/regions included five European countries (i.e., Germany, Italy, Netherlands, England, and France), two Asian countries (i.e., China and Turkey), two North American countries (i.e., the USA and Canada), and one Oceanic country (i.e., Australia).  Figure 4(a) shows that several countries, especially European Union countries, are closely linked together. A total of 2,014 institutions contributed publications on neuropathic pain rehabilitation research. Nine of the top 10 institutions were found in the United States and one is in Canada. Two nonuniversity institutions, namely, the Mayo Clinic and Memorial Sloan Kettering Cancer Center, are in the top 10 institutions and published two ESI Top papers and one ESI Top paper, respectively. The United States, as a developed country, is clearly the leader in this field.

### 4.2. Research Hot Spots and Trends

As an emerging field, rehabilitation medicine has received extensive attention in recent years on account of its important role in SCI, cerebral apoplexy, and osteoarthropathy. We explored emerging topics and concerns in the field of neuropathic pain rehabilitation.

Analysis of the comorbidity map of the references showed that “randomized controlled trials” was the largest cluster, and the other large clusters are as follows:

1. Complex regional pain syndrome: CPRS presents as a type of burning pain and is usually caused by neuropathic pain. Its pathogenesis involves neurogenic inflammation mediated by cytokines and neuropeptides. Studies have shown that spinal cord stimulation, dorsal root ganglion stimulation (DRGS), is effective in treating the disease [39, 40]
2. Spinal cord injury: chronic neuropathic pain after SCI is a complex disease, and transcranial direct current stimulation is effective in clinical treatment [41]. The latest clinical practice guidelines also point to the use of sensors and mechanical devices can help patients achieve functional movement, enhance recovery, and increase neural plasticity, as well as potential adjuncts [42–44]
3. Central sensitization: central sensitization is a kind of hypersensitivity to pain caused by central neural plasticity, which is interwoven with psychoneuroimmunological interactions [34, 35], and is of great significance for the diagnosis and treatment of pain. Recent studies have shown that cGMP-dependent protein kinase I, a nociceptor locator, is a key producer of central sensitization and neuropathic pain [45]. We found that activation of microglia attenuated synaptic transmission and reduced neuroinflammation, synaptic function, and neuralgia. Therefore, chemotherapy offers a potential opportunity to explore microglia function and neuropathic pain treatment [46]

Analysis of the keywords with the strongest citation bursts from 2000 to 2019 revealed major hot spots in the field of neuropathic pain rehabilitation (as shown in Figure 6). The top 25 keywords in 2000 included “reflex sympathetic dystrophy,” “complex regional pain syndrome,” “gabapentin,” “diabetic neuropathy,” and “neuralgia.” The top 25 keywords by the end of 2019 included “exercise” (2014–2019), “functional recovery” (2015–2019), “outcome” (2015–2018), and “questionnaire” (2015–2018). These keywords may predict the frontiers of research as follows:

1. Exercise

In recent years, the idea that exercise is good medicine has been widely accepted by the public, and exercise is among the methods recommended for the treatment of neuropathic pain [47]. Although the mechanism of exercise in improving neuropathic pain has been confirmed in animal experiments, the corresponding mechanism in humans is complex and has not been thoroughly studied.

The effect of sports on the improvement of lower back pain, diabetic neuralgia, and pediatric pain has also been affirmed by professionals [48–50]. Exercise therapy can help patients avoid the adverse effects of drug therapy, relieve pain, and improve their quality of life.

(2) Functional recovery

No evidence from randomized trials indicates that treatment is necessarily effective. For example, randomized clinical trials are needed to determine the efficacy of glucocorticoids or other immunoregulatory therapies in the treatment of neuralgia muscular atrophy [51]. In one experiment, long-term regular exercise was explored as a means to reduce the neuroanalgesic behavior of mice and, ultimately, promote motor function [52]. Another study investigated the efficacy and functional recovery of SCI neuropathic pain symptoms by using long-term intensive locomotor training [53].

(3) Outcome

The efficacy of different interventions in the treatment of neuropathic pain could be evaluated by analyzing data on pain, function, dose, and adverse effects in randomized controlled trials. Knowledge of outcomes can help patients choose the appropriate rehabilitation treatment [54, 55].

### 4.3. Strength and Limitations

This article is the first to summarize the research current status, geographical distribution, research hot spots, and development trends in neuropathic pain rehabilitation worldwide. Our study encompasses 20 years of data extracted from WoS and analyzed by CiteSpace and, thus, provides strong evidence of the future development of research in this field through keywords and subject categories. The soft power of science and technology of each country was visualized using a world map of the distribution of published papers, institutions, journals, and countries. Analysis of the authors and cited authors could help identify leaders in this domain. However, the limitations of our work must be acknowledged. First, although we believe that WoS is a suitably large database that can provide a wide variety of publications critical to our analysis, future researchers could use other databases, such as Scopus, Embase, Ovid-Medline, and China Knowledge Resource Integrated (CNKI), to explore other potential papers. Future studies can broaden the search scope to include more relevant studies to enrich the literature. Finally, some keywords that did not provide much information, such as risk, model, and system, could not be analyzed.

## 5. Conclusion

Our understanding of neuropathic pain rehabilitation has advanced remarkably over the last 20 years. Using bibliometric charts, we illustrated the overall structure of scientific research on neuropathic pain rehabilitation and provided comprehensive information related to this field for other investigators. The most recent burst keywords were “exercise,” “functional recovery,” “outcome,” and “questionnaire.” This analysis provides a comprehensive overview of relevant research conducted in the area of neuropathic pain rehabilitation.

## Figures and Tables

**Table 1:** The top 10 countries of origin of papers in neuropathic pain rehabilitation research.

Country	Papers	Citations WoS	Citations per paper	Open access	*H*-index	ESI top paper
USA	599	19543	32.63	196	71	5
Canada	113	2867	25.37	44	30	0
Peoples R China	92	843	9.16	50	16	0
Germany	91	2134	23.45	20	24	0
Italy	85	2919	34.34	22	25	1
Austria	76	4414	58.08	32	26	1
Netherlands	67	2345	35	37	28	0
England	65	2672	41.11	32	22	1
France	61	2376	38.95	12	19	1
Turkey	60	569	9.48	16	12	0

**Table 2:** The top 10 institutions of origin of papers in neuropathic pain rehabilitation research.

Institutions	Papers	Citations WoS	Citations per paper	Open access	*H*-index	ESI top paper
Univ Toronto	36	980	27.22	12	17	0
Harvard Univ	31	2278	73.48	12	19	1
Univ Washington	27	2042	75.63	10	21	0
Univ Miami	26	698	26.85	14	12	0
Univ Michigan	24	997	41.54	8	14	1
Univ Pittsburgh	22	480	21.82	15	11	0
Washington Univ	22	1086	49.36	11	14	0
Mayo Clin	20	2257	112.85	8	15	2
Mem Sloan Ketteing Canc CTR	19	1098	57.79	7	15	1
Univ British Columbia	19	723	38.05	12	12	0

**Table 3:** The top 10 authors, cocited authors, and cocited references in neuropathic pain rehabilitation research.

Author	Count	Cocited author	Count	Cocited reference	Count
Vera Bril	6	Finnerup NB	113	Attal N, 2010, Eur J Neurol, V17, P1113	22
Run Wang	6	Siddall PJ	95	Kirshblum SC, 2011, J Spinal Cord Med, V34, P547	21
Stefano Tamburin	6	Dworkin RH	90	Treede RD, 2008, Neurology, V70, P1630	21
Julie M Fritz	6	Jensen MP	80	Finnerup NB, 2015, Lancet Neurol, V14, P162	20
J J Labat	5	Woolf CJ	64	Haanpaa M, 2011, Pain, V152, P14	20
R Robert	5	Attal N	63	Woolf CJ, 2011, Pain, V152, P0	20
N B FInnerup	5	Bouhassira D	60	Dworkin RH, 2007, Pain, V132, P237	14
T Riant	5	Melzack R	58	Mulhall JP, 2008, J Sex Med, V5, P1126	14
B C Craven	4	Baron R	50	Backonja M, 1998, Jama-J AM MED Assoc, V280, P1831	12
A Townson	4	Harden RN	48	Van de Vusse AC, 2004, BMC Neurol, V4, P0	12

**Table 4:** The top 10 journal of origin of papers in the neuropathic pain rehabilitation research.

Journals	Papers	Citations WoS	Citations per paper	WoS categories	IF 2019	Quartile	*H*-index
Archives of Physical Medicine and Rehabilitation	128	4031	31.49	Rehabilitation; sport sciences	3.098	Q1; Q1	37
Spinal Cord	34	1024	30.12	Clinical neurology; rehabilitation	1.773	Q3; Q2	14
American Journal of Physical Medicine Rehabilitation	27	593	21.96	Rehabilitation; sport sciences	1.838	Q2; Q3	10
European Journal of Physical and Rehabilitation Medicine	23	240	10.43	Rehabilitation	2.258	Q1	9
Physical Therapy	23	1823	79.26	Orthopedics; rehabilitation	3.14	Q1; Q1	13
Journal of Rehabilitation Research and Development	20	554	27.7	Rehabilitation (SSCI); rehabilitation (SCIE)	1.277 (2016)	Q2; Q3	16
Neurorehabilitation and Neural Repair	18	614	34.11	Clinical neurology; rehabilitation	3.982	Q1; Q1	10
PM R	18	347	19.28	Rehabilitation; sport sciences	1.821	Q2; Q3	8
Journal of Sexual Medicine	16	505	31.56	Urology and nephrology	3.293	Q2	10
Pain Medicine	16	135	8.44	Anesthesiology; medicine, general and internal	2.513	Q2; Q2	8

## Data Availability

All research data used to support the findings of this study are included within the article.
